# Gold Nanocluster–Amino Acid Interactions: Assessment of DFTB with Dispersion Corrections

**DOI:** 10.1021/acsomega.5c09355

**Published:** 2026-02-11

**Authors:** Jerhett Morehouse, Alyssa McPhee, Emily Howie, Luiz F. L. Oliveira

**Affiliations:** 5663 Mount Vernon Nazarene University, Chemistry and Physics Program, Mount Vernon, 43050 Ohio, United States

## Abstract

Understanding the interaction between gold nanostructures and biomolecules is critical for advancing applications in nanomedicine, biosensing, and bioelectronics. Here, we assess the performance of density functional tight binding (DFTB) with Grimme’s D3­(BJ) dispersion correction by comparing it to available density functional theory (DFT) results in the literature for gold nanocluster–amino acid complexes. Five clusters (Au_3_, Au_8_, Au_13_, Au_20_, Au_32_) interacting with ten amino acids were investigated at both amine and carboxyl binding sites. System selection was guided by the availability of the corresponding DFT results in the literature. DFTB reproduces the qualitative binding preference for amine over carboxyl adsorption, with interaction energies typically within 2–3 kcal/mol of DFT for Au_3_ and Au_8_. Au–X bond lengths are systematically longer by ∼0.4–0.7 Å, and larger deviations appear for Au_13_ and nitrogen-containing cyclic amino acids. For Au_20_, good agreement with DFT is obtained for alanine at the amine binding site and tryptophan at both the amine and carboxyl sites, suggesting that DFTB remains reliable beyond the smallest clusters. For Au_32_, although a direct comparison is not possible, the data indicate similar binding preferences to those found for smaller clusters. In addition, nitrogen-containing cyclic amino acids (such as histidine, proline, and tryptophan) tend to show larger discrepancies, reflecting the increased importance of polarization, charge redistribution, and multiple competing binding motifs in these systems. As the cluster size increases from Au_3_ to Au_32_, DFTB generally preserves qualitative trends but exhibits growing quantitative deviations, indicating that transferability toward more metallic, nanoparticle-like regimes must be assessed with caution. Our results demonstrate that DFTB + D3­(BJ) provides an efficient and sufficiently accurate framework for screening Au–biomolecule interactions.

## Graphical abstract

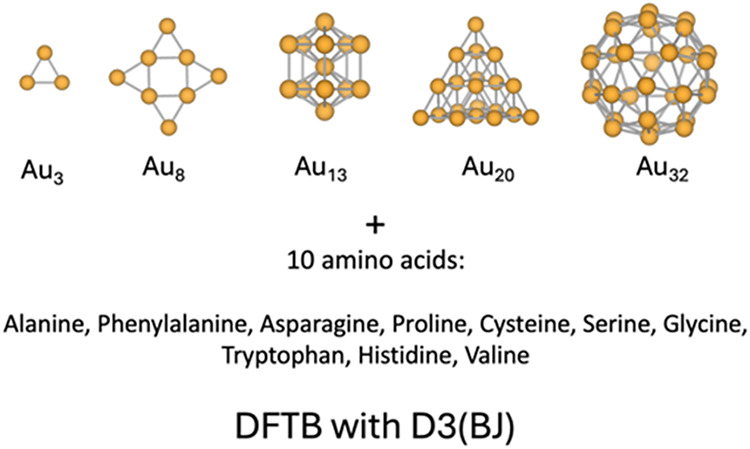

## Introduction

The interactions of biomolecules and nanomaterials play a central role in applications ranging from nanomedicine, biosensing, bioelectronics, and bioelectrochemistry.^1–4^ Among these, gold nanoclusters (AuNCs) and nanoparticles (AuNPs) exhibit distinctive optical, electronic, and catalytic properties.^2,5–9^ Their biocompatibility and tunable surface chemistry have made them promising candidates for biomedical applications, including use as drug carriers and contrast agents.^10–12^ There are still challenges to making the biomedical applications of these materials more widely used.^13^ A few examples are improving the stability of AuNPs in biological applications, understanding the impact of size and shape on the performance of AuNPs as drug carriers, and understanding how functionalization can enhance their interaction with biomolecules. To overcome such challenges, a deep understanding of the physicochemical processes related to the interaction of AuNPs with biomolecules at the molecular level is crucial.

Theoretical studies have successfully contributed to these endeavors.^2,6,14–27^ Such success is mainly due to advances in computational chemistry and the rapid increase in the available computational power. Density functional theory (DFT) remains the primary tool for modeling such interactions. Although this level of theory is successful in describing small to medium-sized clusters, it becomes computationally unfeasible for larger systems due to its computational cost. An effective approach to address this is to use a parametrized quantum mechanical method such as density functional tight binding (DFTB).^28–30^ DFTB offers an efficient compromise between computational cost and accuracy by retaining an explicit quantum description of the electronic structure, making it especially well-suited for large, complex molecular systems, including interactions between AuNCs and AuNPs with biomolecules.

**Figure 1:**
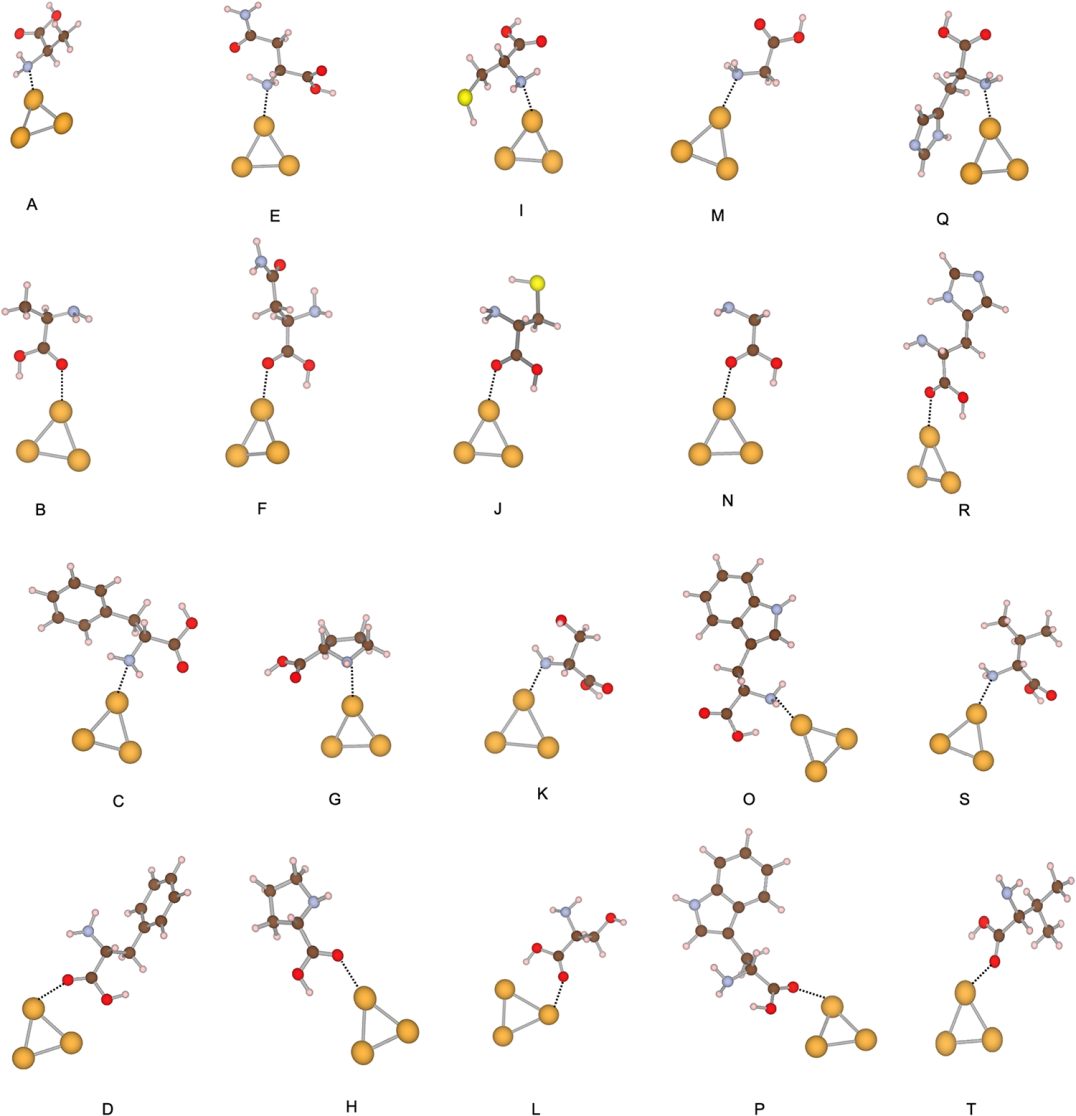
Local minima of the Au_3_ systems. The amino acids in each structure and interacting side are (A) alanine, amine site; (B) alanine, carboxylic site; (C) phenylalanine, amine site; (D) phenylalanine, carboxylic site; (E) asparagine, amine site; (F) asparagine, carboxylic site; (G) proline, amine site; (H) proline, carboxylic site; (I) cysteine, amine site; (J) cysteine, carboxylic site; (K) serine, amine site; (L) serine, carboxylic site; (M) glycine, amine site; (N) glycine, carboxylic site; (O) tryptophan, amine site; (P) tryptophan, carboxylic site; (Q) histidine, amine site; (R) histidine, carboxylic site; (S) valine, amine site; (T) valine, carboxylic site. Gold atoms are represented in yellow, oxygen atoms in red, sulfur atoms in light yellow, hydrogen atoms in light pink, carbon atoms in brown, and nitrogen atoms in steel blue.

**Figure 2:**
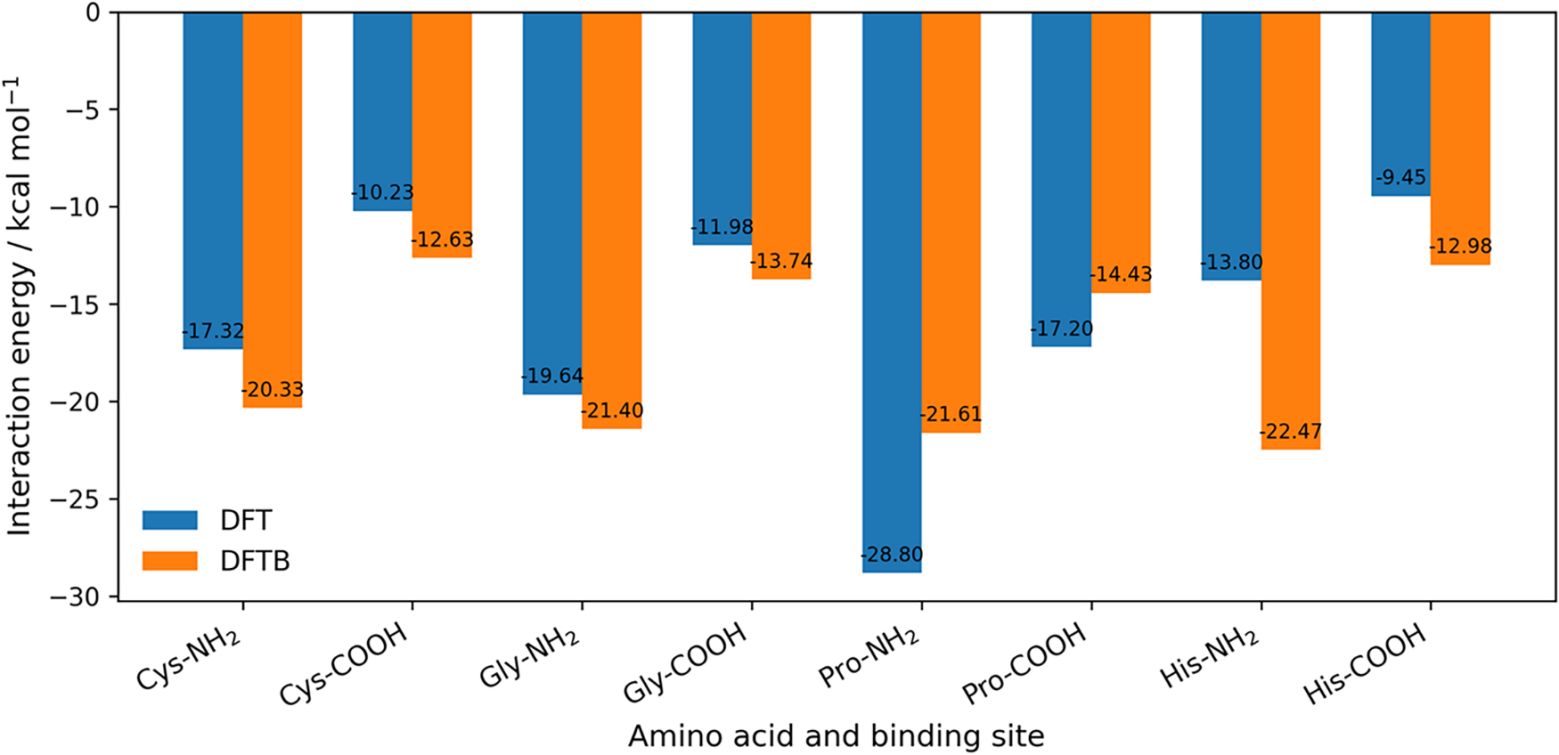
Comparison of DFTB and DFT interaction energies for Au_3_–amino acid complexes at the amine (NH_2_) and carboxyl (COOH) binding sites. Bars show the adsorption energies obtained with DFTB (orange) and DFT (blue) for cysteine, glycine, proline, and histidine, the amino acids for which DFT reference values are available for the Au_3_ cluster.

**Table 1:** Interaction Energies (*E*_int_, kcal/mol) and Au–X Bond Llengths (d, Å) for Au_3_–Amino Acid Complexes at Amine (X = N) and Carboxylic (X = O) Sites, as Obtained from DFTB Calculations

complex, interacting site	*E*_int_ (kcal/mol)	*d* (Au-X) (Å)
Au_3_-alanine, amine site	–23.70	2.31
Au_3_-alanine, carboxylic site	–14.07	2.35
Au_3_-asparagine, amine site	–25.24	2.31
Au_3_-asparagine, carboxylic site	–15.95	2.33
Au_3_-cysteine, amine site	–20.33	2.33
Au_3_-cysteine, carboxylic site	–12.63	2.36
Au_3_-glycine, amine site	–21.40	2.31
Au_3_-glycine, carboxylic site	–13.74	2.35
Au_3_-histidine, amine site	–22.47	2.33
Au_3_-histidine, carboxylic site	–12.98	2.36
Au_3_-phenylalanine, amine site	–24.14	2.19
Au_3_ -phenylalanine, carboxylic site	–13.48	2.30
Au_3_-proline, amine site	–21.61	2.41
Au_3_-proline, carboxylic site	–14.43	2.35
Au_3_-serine, amine site	–23.62	2.18
Au_3_-serine, carboxylic site	–14.75	2.28
Au_3_-tryptophan, amine site	–19.17	2.13
Au_3_-tryptophan, carboxylic site	–14.71	2.24
Au_3_-valine, amine site	–21.77	2.28
Au_3_-valine, carboxylic site	–14.59	2.22

**Figure 3:**
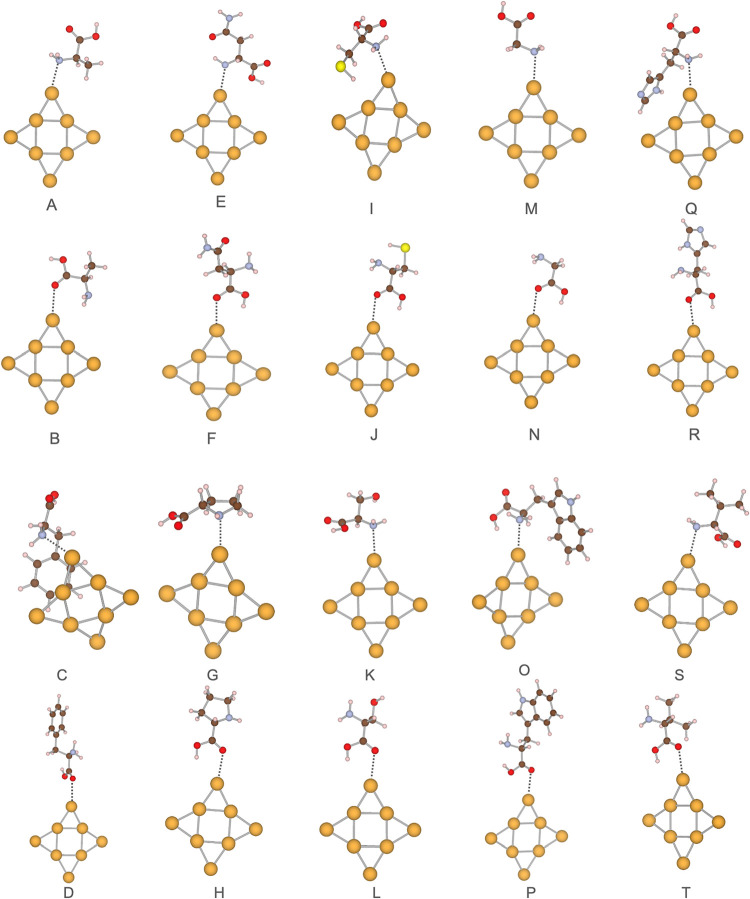
Local minima of the Au_8_ systems. The amino acids in each structure and interacting side are (A) alanine, amine site; (B) alanine, carboxylic site; (C) phenylalanine, amine site; (D) phenylalanine, carboxylic site; (E) asparagine, amine site; (F) asparagine, carboxylic site; (G) proline, amine site; (H) proline, carboxylic site; (I) cysteine, amine site; (J) cysteine, carboxylic site; (K) serine, amine site; (L) serine, carboxylic site; (M) glycine, amine site; (N) glycine, carboxylic site; (O) tryptophan, amine site; (P) tryptophan, carboxylic site; (Q) histidine, amine site; (R) histidine, carboxylic site; (S) valine, amine site; (T) valine, carboxylic site. Gold atoms are represented in yellow, oxygen atoms in red, sulfur atoms in light yellow, hydrogen atoms in light pink, carbon atoms in brown, and nitrogen atoms in steel blue.

**Figure 4:**
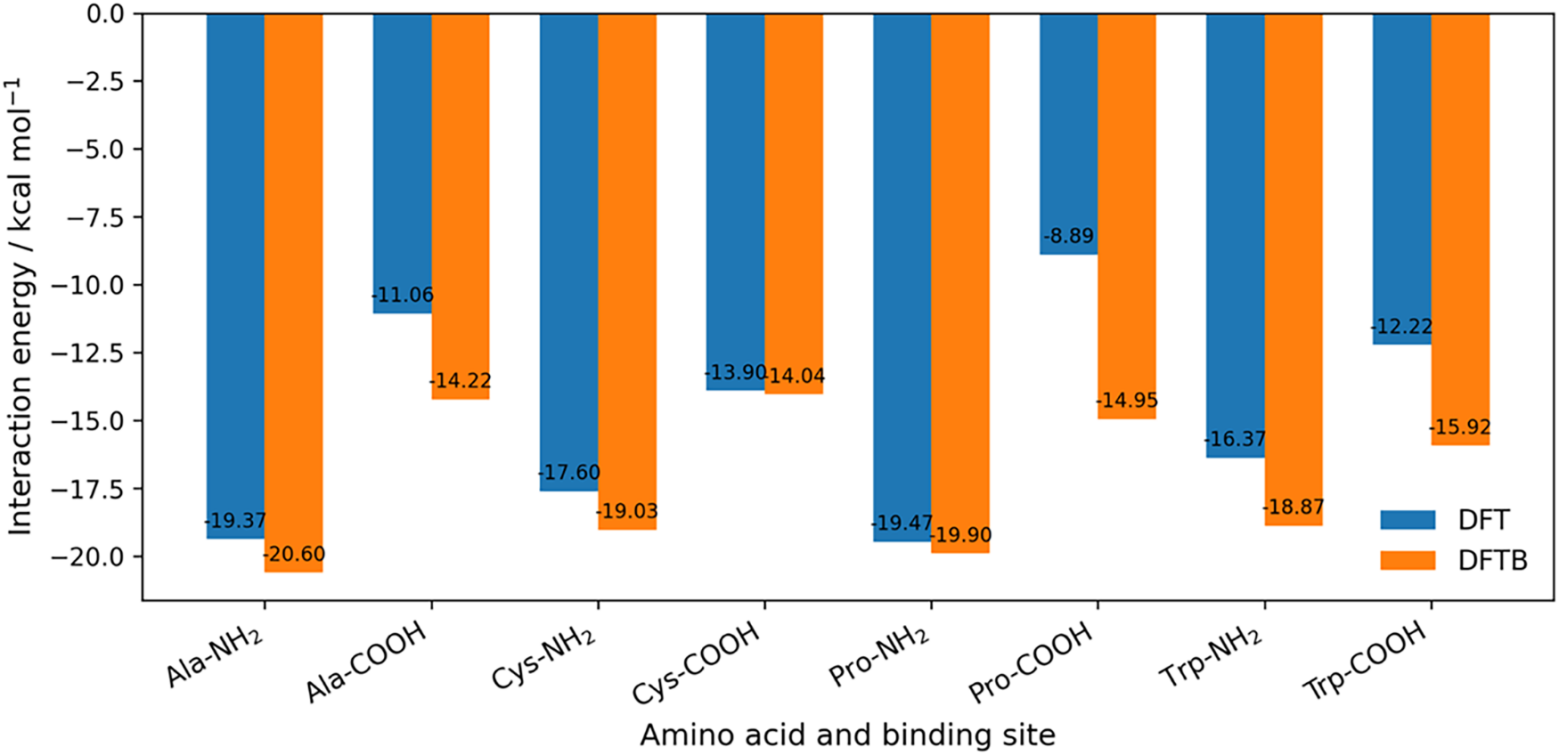
Comparison of DFTB and DFT interaction energies for Au_8_–amino acid complexes at the amine (NH_2_) and carboxyl (COOH) binding sites. Bars show the adsorption energies obtained with DFTB (orange) and DFT (blue) for alanine, cysteine, proline, and tryptophan, the amino acids for which DFT reference values are available for the Au_8_ cluster.

**Table 2:** Interaction Energies (*E*_int_, kcal/mol) and Au–X Bond Lengths (d, Å) for Au_8_–Amino Acid Complexes at Amine (X = N) and Carboxylic (X = O) Sites, as Obtained from DFTB Calculations

complex, interacting site	*E*_int_ (kcal/mol)	*d* (Au-X) (Å)
Au_8_-alanine, amine site	–20.60	2.76
Au_8_-alanine, carboxylic site	–14.22	2.92
Au_8_-asparagine, amine site	–22.81	2.72
Au_8_-asparagine, carboxylic site	–16.40	2.83
Au_8_-cysteine, amine site	–19.03	2.81
Au_8_-cysteine, carboxylic site	–14.04	2.87
Au_8_-glycine, amine site	–19.27	2.74
Au_8_-glycine, carboxylic site	–14.62	2.85
Au_8_-histidine, amine site	–23.40	2.82
Au_8_-histidine, carboxylic site	–14.09	2.86
Au_8_-phenylalanine, amine site	–26.22	2.85
Au_8_-phenylalanine, carboxylic site	–15.10	2.85
Au_8_-proline, amine site	–19.90	2.79
Au_8_-proline, carboxylic site	–14.95	2.83
Au_8_-serine, amine site	–20.90	2.73
Au_8_-serine, carboxylic site	–15.11	2.85
Au_8_-tryptophan, amine site	–18.87	2.75
Au_8_-tryptophan, carboxylic site	–15.92	2.83
Au_8_-valine, amine site	–20.07	2.79
Au_8_-valine, carboxylic site	–15.11	2.83

The method relies on a set of parameters for each atomic pair in the system. These parameters include two-center integrals and repulsive potentials, which are derived directly from DFT reference calculations rather than fitted to empirical data. This first-principles foundation gives DFTB a significant advantage over empirical force fields and traditional semiempirical methods such as AM1 or PM3, in terms of both transferability and accuracy.

DFTB parameter sets are frequently tailored to specific applications yet often retain good performance across related systems. This combination of transferability, efficiency, and accuracy makes DFTB a practical method for simulating Au–biomolecule interactions.

DFTB has been applied successfully to a wide range of systems and processes, including pristine AuNCs and AuNPs,^31–33^ biological systems,^34,35^ molecular clusters,^36,37^ and chemical reactions.^38–40^ However, despite its demonstrated potential, only a few studies have used DFTB to investigate the interaction of AuNPs with biomolecules. To the best of our knowledge, only two such studies exist in the literature. In the most recent one,^41^ DFTB was used to provide optimized structures of gold chains with varying atomic lengths in realistic DNA environments. The other study found was performed by Dominguez-Castro et al.^35^ in which they studied the Au_3_–Proline and compared it with the DFT calculations published by Rai et al.^17^ Supported by the good agreement, they went on and studied other more complex systems. Even though this work represents an important step in the study of AuNCs and biomolecules through DFTB, only Au_3_-proline was directly compared against DFT data. Additionally, dispersion was included via a Lennard-Jones potential; however, more modern dispersion correction methods are now available and offer greater accuracy. Thus, a broader range of reference systems combined with a more accurate dispersion is needed.

To address this gap, in this paper, we present an energetic and structural analysis of selected AuNCs interacting with amino acids. Our study includes Grimme’s D3 ^42^ dispersion corrections with Becke–Johnson damping (D3­(BJ)),^43^ and we present results of adsorption energies and adsorption distances. The systems were selected based on the availability of comparative DFT data in the literature. Specifically, we investigated five gold clusters, namely Au_3_, Au_8_, Au_13_, Au_20_, and Au_32_, in interaction with a representative set of ten amino acids: asparagine, glycine, proline, tryptophan, alanine, cysteine, histidine, phenylalanine, serine, and valine. These systems span a range of gold nanocluster sizes and biomolecular types, enabling a representative evaluation of trends in adsorption behavior. Moreover, we considered two binding modes to capture different adsorption configurations. The corresponding DFT levels of theory (functional and basis set) used in those reference studies are provided later in the text within the discussion of each cluster.

While DFTB has clear advantages in efficiency and retains an explicit electronic structure description, it also has intrinsic limitations that are particularly relevant for metal–biomolecule interfaces. The method relies on parametrization largely based on small molecules and clusters, so phenomena involving strong charge transfer, enhanced polarization, or highly delocalized metallic states may be only approximately captured. As a result, good performance can be expected for small to medium-sized Au clusters, whereas quantitative transferability to larger, more metallic nanoparticles must be critically assessed against higher-level benchmarks.

In the next section, we present the computational strategy employed to investigate the adsorption of biomolecules on small gold nanoclusters, including the parameters and methods used within the DFTB framework. We then describe and analyze the resulting adsorption structures, comparing them to available data from DFT calculations. This is followed by a discussion of the results in the context of accuracy, efficiency, and transferability. The paper concludes with a summary of key findings and suggestions for future directions.

## Methods

All DFTB calculations presented were performed using the DFTB+ code, version 22.2.^44^ Initial structures were constructed manually in Avogadro^45^ using well-established structural motifs reported for gold clusters. Geometry optimizations used a rational function optimizer with a maximum of 1000 steps. The convergence criterion for the forces during geometry optimization was set to 10^–7^ a.u., while the threshold for charge variation in the SCC procedure was set to 10^–7^ a.u. Dispersion interactions were included via Grimme’s D3 with Becke–Johnson damping (D3­(BJ)).^43^ Alternative dispersion schemes for DFTB exist, such as the charge-dependent correction by Petraglia et al.,^46^ but these were not employed here. The Slater–Koster^47^ parameters were taken from the *auorg-1–1 set* ^48,49^ set for Au–X pairs (X = H, C, N, O, S) and the *mio-1–1 set* ^28^ for light-element pairs. The *auorg-1–1* parameters are derived from scalar-relativistic DFT reference calculations; therefore, the dominant relativistic effects for gold are included at the level of the underlying Slater–Koster files, whereas explicit spin–orbit coupling is not treated in the present DFTB calculations. Calculations were performed using a spin-polarized electronic procedure, and spin constants were specified for all elements present (H, C, N, O, S, Au).

The interaction energy is computed as follows

1 $$E_{\mathrm{int}}=E_{\mathrm{complex}}-\left(E_{\mathrm{aminoacid}}+E_{\mathrm{Au},c\mathrm{luste}r}\right)$$

where *E*_complex_, *E*_amino acid_, and *E*_Au,cluster_ are the total energies of the relaxed systems composed of the complex, of the isolated amino acid, and of the gold cluster, respectively.

## Results and Discussion

The structures presented in this study were selected from previous works in which DFT calculations were used. Alternative structural motifs exist for small gold clusters; for instance, Au_8_ can adopt both planar and three-dimensional geometries. The present study, however, employs representative low-energy configurations reported in the literature. These structures are sufficient for the purposes of assessing the performance of DFTB relative to the DFT calculation and for capturing general adsorption trends. The results are presented in the following subsections, grouped by the five gold clusters investigated. More negative values of the adsorption energy indicate stronger interactions between the adsorbate and cluster and thus correspond to more thermodynamically stable adsorbed configurations. All molecular structures in this work are rendered using Visualization for Electronic and STructural Analysis (VESTA).^50^

### Au_3_ – Amino Acid Systems

The lowest-energy structures of the Au_3_–amino acid complexes are shown in Figure 1, with interaction energies and bond lengths summarized in Table 1. DFTB predicts adsorption energies of −19.17 to −25.24 kcal/mol at the amine site and −12.63 to −15.95 kcal/mol at the carboxyl site. For cysteine, DFTB gives −20.33 kcal/mol at the amine site and −12.63 kcal/mol at the carboxyl site, compared with DFT values of −17.32 and −10.23 kcal/mol, respectively.^18^ For glycine, the corresponding values are −21.40 and −13.74 kcal/mol with DFTB, versus −19.64 and −11.98 kcal/mol from DFT.^18^ In the case of proline, DFTB predicts −21.61 kcal/mol at the amine site and −14.43 kcal/mol at the carboxyl site, while DFT calculations give −36.45 and −28.80 kcal/mol at the amine site and −17.20 and −24.66 kcal/mol at the carboxyl site.^17^ The two DFT values reported for proline were obtained with different basis set levels. For histidine, DFTB yields −22.47 kcal/mol at the amine site and −12.98 kcal/mol at the carboxyl site, whereas the corresponding DFT values are −13.80 and −9.45 kcal/mol.^19^ These results, summarized in Figure 2, indicate that DFTB generally reproduces the DFT interaction energies within 2–3 kcal/mol, except for nitrogen-containing cyclic systems, which remain more difficult to describe accurately. This behavior is consistent with the more complex electronic structure of heterocycles and ring-constrained backbones, where stronger polarization, partial charge transfer, and multiple competing adsorption motifs challenge the simpler charge and polarization model used in DFTB. The DFT reference values were taken from studies that employed the B3LYP functional with LANL2DZ-type relativistic effective core potentials for gold and polarized basis sets (6–31+G to 6–311++G) for the amino-acid atoms.

In terms of geometry, DFTB predicts Au–X (X = N,O) bond lengths of 2.18–2.41 Å for amine coordination and 2.22–2.36 Å for carboxyl coordination. For cysteine, DFTB gives 2.33 Å at the amine site and 2.36 Å at the carboxyl site, compared with DFT values of 2.22 Å and 2.25 Å, respectively.^16,18^ For glycine, the corresponding DFTB values are 2.31 Å at the amine site and 2.35 Å at the carboxyl site, while DFT predicts 2.21–2.22 Å and 2.26–2.30 Å.^16,18^ In histidine, DFTB yields 2.33 Å for the Au–N bond and 2.36 Å for the Au–O bond, slightly longer than the DFT results of 2.26 Å and 2.22 Å.^19^ Proline shows larger differences, with DFTB predicting 2.41 Å at the amine site and 2.35 Å at the carboxyl site, compared with DFT values of 2.20–2.21 Å and 2.24–2.25 Å.^17^For the other amino acids considered (alanine, asparagine, phenylalanine, serine, tryptophan, and valine), no DFT reference values are available, but DFTB predicts Au–N and Au–O bond lengths in the ranges 2.13–2.31 Å and 2.22–2.35 Å, respectively. Overall, DFTB tends to overestimate Au–X bond lengths by approximately 0.1–0.2 Å relative to DFT, with the largest discrepancies observed for proline.

### Au_8_ – Amino Acid Systems

For the Au_8_–amino acid complexes, the lowest-energy structures are shown in Figure 3, with interaction energies and bond lengths summarized in Table 2. DFTB predicts adsorption energies between −19.03 and −26.22 kcal/mol at the amine site and −14.04 and −16.40 kcal/mol at the carboxyl site, consistently favoring amine coordination across all amino acids. Where DFT reference data are available, the agreement is generally within 1–3 kcal/mol. For alanine, DFTB gives −20.60 kcal/mol at the amine site and −14.22 kcal/mol at the carboxyl site, while DFT (B3LYP functional, LANL2DZ basis set for Au, and 6–31G­(d,p) basis set for nonmetals) yields −19.37 and −11.06 kcal/mol, respectively.^22^ For cysteine, DFTB predicts −19.03 kcal/mol at the amine site and −14.04 kcal/mol at the carboxyl site, compared with DFT results obtained using the PBE functional with a cc-pVTZ-PP basis set for Au and a cc-pVTZ basis set for the nonmetal atoms, which give −17.60 and −13.90 kcal/mol,^21^ respectively. Proline shows good agreement at the amine site, with DFTB giving −19.90 kcal/mol and DFT (PBE1PBE functional, SDD basis set for Au, 6–311++G basis set for nonmetals) −19.47 kcal/mol.^51^ Tryptophan binds with −18.87 kcal/mol at the amine site and −15.92 kcal/mol at the carboxyl site in DFTB, compared with −16.37 and −12.22 kcal/mol from DFT.^22^ Larger discrepancies appear for proline at the carboxyl site, where DFTB gives −14.95 kcal/mol and DFT −8.89 kcal/mol, a difference of 6.06 kcal/mol, and for tryptophan at the carboxyl site, −12.22 kcal/mol^22^ from DFT calculations, giving a deviation of 3.70 kcal/mol. For asparagine, glycine, histidine, phenylalanine, serine, and valine, only the DFTB results are available, emphasizing the need for additional benchmarking data. The comparison between DFT and DFTB is summarized in Figure 4.

The corresponding Au–X bond lengths follow the same trend. DFTB predicts Au–N distances of 2.72–2.85 Å and Au–O distances of 2.83–2.92 Å, systematically longer than reported DFT values of 2.23–2.44 Å by about 0.40–0.60 Å. For alanine, DFTB gives 2.76 Å at the amine site and 2.92 Å at the carboxyl site, while DFT values are 2.27 and 2.36 Å.^22^ For cysteine, the Au–N and Au–O distances are 2.81 and 2.87 Å in DFTB compared with 2.23 and 2.27 Å in DFT.^21^ For tryptophan, DFTB predicts 2.75 and 2.83 Å, whereas DFT gives 2.25 and 2.44 Å.^22^ Although absolute bond lengths are systematically overestimated, the relative site preference is preserved, with amine bonds generally shorter than carboxyl bonds.

The results of the Au_8_–amino acid systems demonstrate that DFTB qualitatively reproduces the preferred binding mode and relative interaction strengths of amino acids on this cluster, although systematic overestimation of bond lengths and moderate deviations in interaction energies are observed. In this size regime, the binding is dominated by local Au–X coordination, with dispersion interactions providing an additional stabilization that is more pronounced for larger and more polarizable side chains, which D3­(BJ) accounts for in an approximate but computationally efficient way.

### Au_13_ – Amino Acid Systems

For the Au_13_–amino acid complexes, Figure 5, DFTB predicts a wide range of adsorption energies, spanning from moderate stabilization to apparent overstabilization in certain cases, Table 3. Alanine binds with −16.38 and −5.21 kcal/mol at the amine and carboxyl sites, while cysteine shows −14.07 and −6.75 kcal/mol at the respective sites. Serine (−15.84 and −5.85 kcal/mol) and valine (−12.35 and −8.13 kcal/mol) all exhibit binding strengths consistent with weak to moderate adsorption. In contrast, glycine and phenylalanine show unusually large values with adsorption energies of −39.0 and −47.2 kcal/mol at the amine site, respectively, and similarly strong stabilization at the carboxyl site. These highly negative energies are accompanied by pronounced deformation of the Au_13_ cluster during optimization, suggesting that its structural flexibility plays a key role in the observed binding patterns.

**Figure 5:**
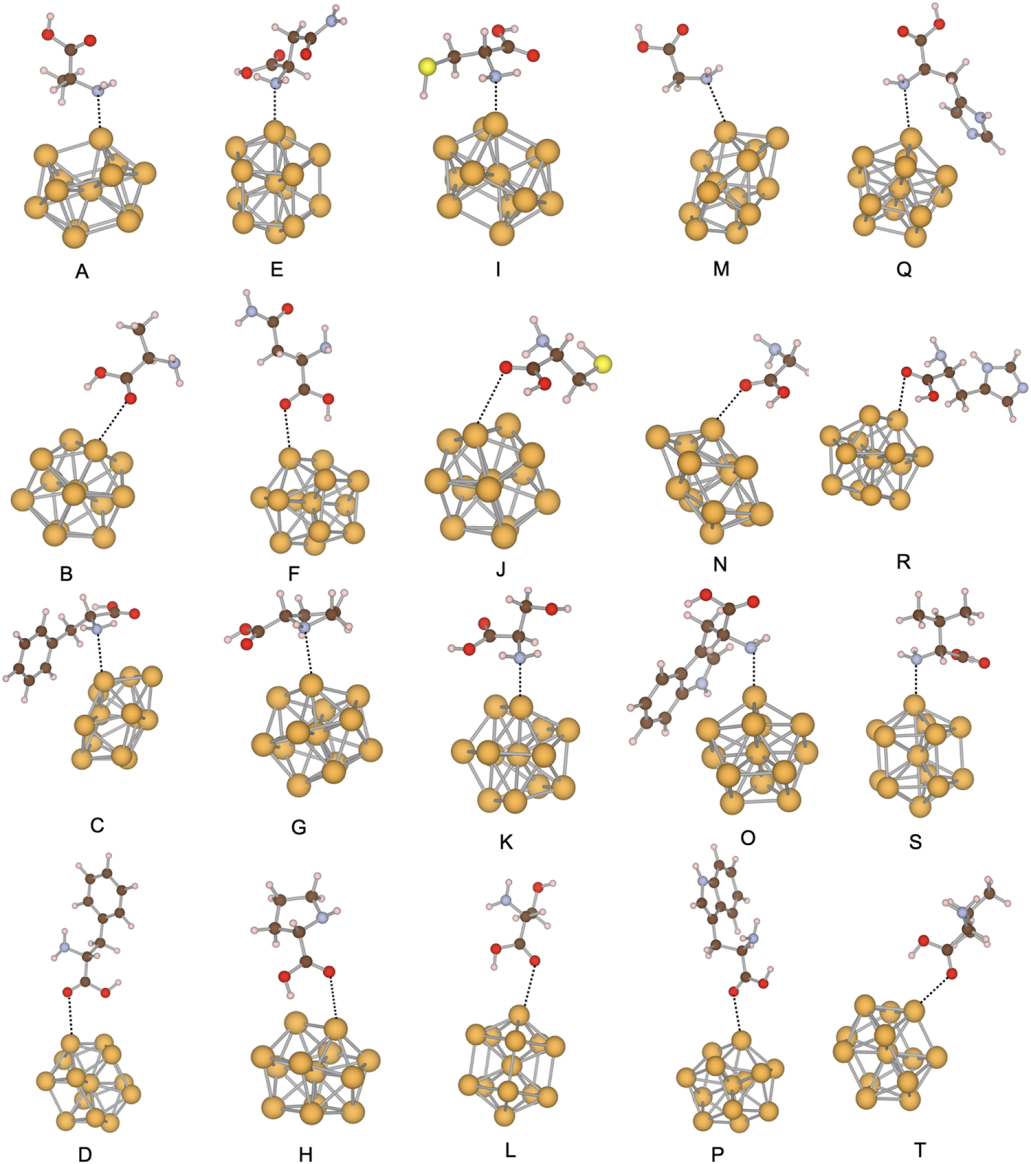
Local minima structures of the Au_13_ systems. The amino acids in each structure and interacting side are (A) alanine, amine site; (B) alanine, carboxylic site; (C) phenylalanine, amine site; (D) phenylalanine, carboxylic site; (E) asparagine, amine site; (F) asparagine, carboxylic site; (G) proline, amine site; (H) proline, carboxylic site; (I) cysteine, amine site; (J) cysteine, carboxylic site; (K) serine, amine site; (L) serine, carboxylic site; (M) glycine, amine site; (N) glycine, carboxylic site; (O) tryptophan, amine site; (P) tryptophan, carboxylic site; (Q) histidine, amine site; (R) histidine, carboxylic site; (S) valine, amine site; (T) valine, carboxylic site. Gold atoms are represented in yellow, oxygen atoms in red, sulfur atoms in light yellow, hydrogen atoms in light pink, carbon atoms in brown, and nitrogen atoms in steel blue.

**Table 3:** Interaction Energies (*E*_int_, kcal/mol) and Au–X Bond Lengths (d, Å) for Au_13_–Amino Acid Complexes at Amine (X = N) and Carboxylic (X = O) Sites, as Obtained from DFTB Calculations

complex, interacting site	*E*_int_ (kcal/mol)	*d* (Au-X) (Å)
Au_13_-alanine, amine site	–16.38	2.36
Au_13_-alanine, carboxylic site	–5.21	3.09
Au_13_-asparagine, amine site	–9.63	2.38
Au_13_-asparagine, carboxylic site	–16.29	2.80
Au_13_-cysteine, amine site	–14.07	2.41
Au_13_-cysteine, carboxylic site	–6.75	3.31
Au_13_-glycine, amine site	–39.01	2.82
Au_13_-glycine, carboxylic site	–36.00	3.01
Au_13_-histidine, amine site	–20.18	2.83
Au_13_-histidine, carboxylic site	–8.60	3.08
Au_13_-phenylalanine, amine site	–47.18	2.87
Au_13_ -phenylalanine, carboxylic site	–6.40	3.07
Au_13_-proline, amine site	–16.57	2.81
Au_13_-proline, carboxylic site	–8.42	2.83
Au_13_-serine, amine site	–15.84	2.38
Au_13_-serine, carboxylic site	–5.85	3.05
Au_13_-tryptophan, amine site	–23.83	2.36
Au_13_-tryptophan, carboxylic site	–8.75	2.83
Au_13_-valine, amine site	–12.35	2.41
Au_13_-valine, carboxylic site	–8.13	2.79

Bond-length analysis supports this interpretation. Au–X distances fall in the range of 2.36–2.87 Å for amine coordination and 2.79–3.31 Å for carboxylic coordination, following the general trend of shorter Au–N versus longer Au–O bonds. Alanine and proline exhibit amine-site distances of 2.36 to 2.81 Å, while their carboxyl sites extend to 2.83–3.09 Å. Cysteine shows one of the longest Au–O contacts at 3.31 Å, consistent with weaker coordination at the carboxyl group. Where DFT benchmarks are available, proline shows good agreement, with deviations of only ∼2 kcal/mol in energy and ∼0.1–0.2 Å in bond length.^51^

Interestingly, this variability aligns with previous reports that Au_13_ clusters are structurally flexible and sensitive to their chemical environment. Shahnazari and co-workers^14^ demonstrated that Au_13_ undergoes significant structural and bonding rearrangements when interacting with nucleobases, a behavior also evident here in the exaggerated stabilization of glycine and phenylalanine. This suggests that Au_13_ may be especially prone to structural distortion upon adsorption, making it a challenging system for consistent benchmarking. More generally, Au_13_ marks the onset of stronger structural flexibility, and the associated ligand-induced reconstructions amplify the sensitivity of adsorption energies to the underlying approximations of DFTB. In this context, it is worth noting that for related Pt clusters, it has been shown that different morphologies are not always ranked in the same order by DFT and DFTB,^52^ underscoring that structurally flexible cluster sizes such as Au_13_ are particularly demanding test cases for any approximate electronic-structure method.

### Au_20_ – Amino Acid Systems

For the Au_20_–amino acid complexes, Figure 6, DFTB predicts adsorption energies ranging from −8.09 to −23.69 kcal/mol at the amine site and from −4.59 to −8.44 kcal/mol at the carboxyl site. The interaction energies and bond lengths for these systems are given in Table 4. The strongest stabilization occurs for alanine with −23.69 kcal/mol at the amine site (−5.30 kcal/mol at the carboxyl site), and for phenylalanine, with −18.98 and −6.20 kcal/mol. Weaker interactions are observed for glycine, which binds with −8.62 and −5.05 kcal/mol, and for tryptophan, which binds with −8.09 and −8.44 kcal/mol. Comparison with available DFT results^22^ (B3LYP functional, LANL2DZ basis set for Au, and 6–31G­(d,p) basis set for nonmetals) highlights mixed performance. For alanine, DFT reports −14.97 kcal/mol at the amine site and −6.46 kcal/mol at the carboxyl site. DFTB therefore overestimates the amine binding by nearly 10 kcal/mol while reproducing the carboxyl site within ∼1 kcal/mol. For tryptophan, DFT values of −11.30 and −5.07 kcal/mol are reported for the amine and carboxyl sites, respectively. In this case, DFTB underestimates amine stabilization by 3.21 kcal/mol and overestimates carboxyl binding by 3.37 kcal/mol. For the other amino acids, asparagine, cysteine, glycine, histidine, proline, serine, and valine, no DFT data are available, and only the DFTB predictions are reported. Overall, DFTB consistently captures the preference for amine over carboxyl binding but tends to overbind at the amine group relative to DFT, Figure 7.

**Figure 6:**
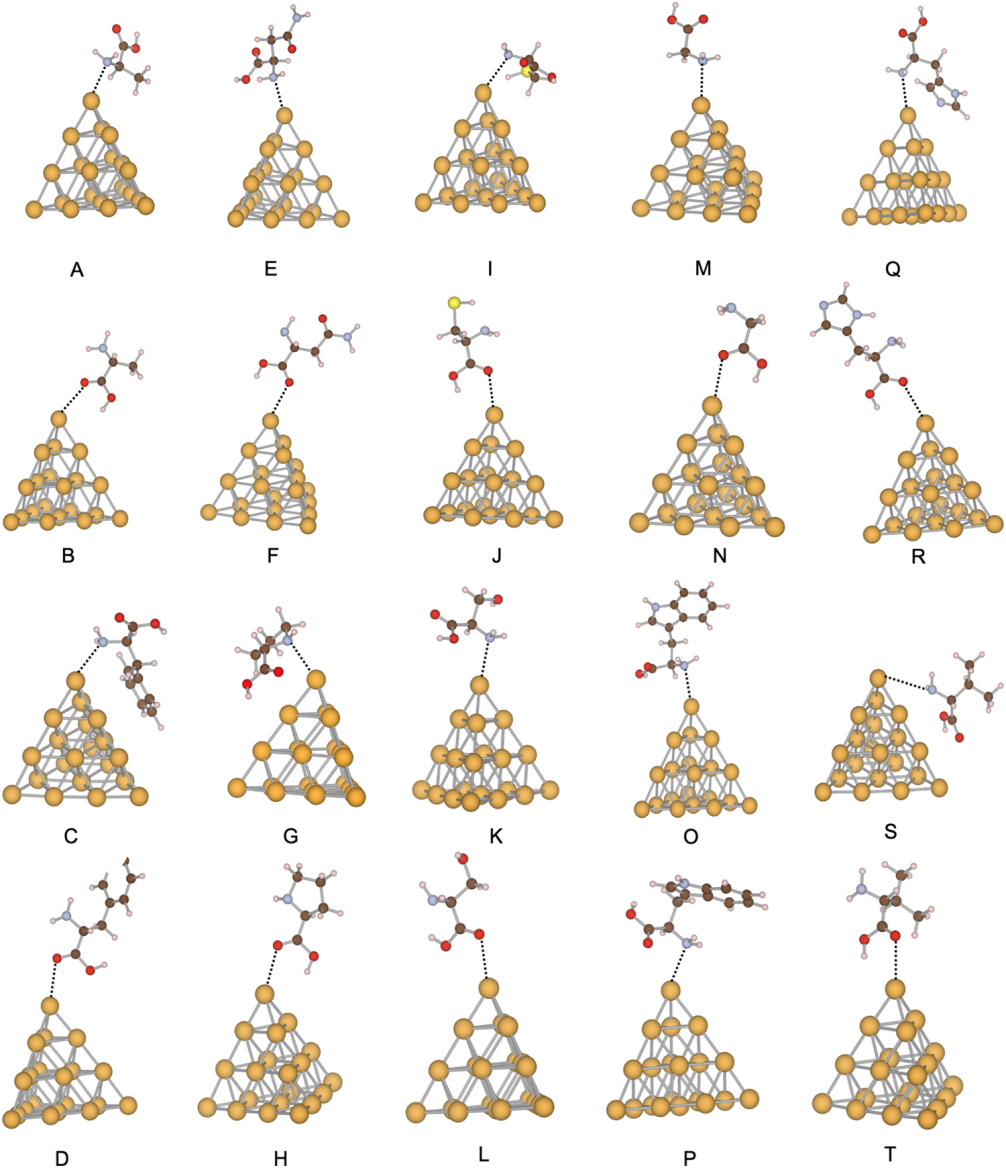
Local minima structures of the Au_20_ systems. The amino acids in each structure and interacting side are (A) alanine, amine site; (B) alanine, carboxylic site; (C) phenylalanine, amine site; (D) phenylalanine, carboxylic site; (E) asparagine, amine site; (F) asparagine, carboxylic site; (G) proline, amine site; (H) proline, carboxylic site; (I) cysteine, amine site; (J) cysteine, carboxylic site; (K) serine, amine site; (L) serine, carboxylic site; (M) glycine, amine site; (N) glycine, carboxylic site; (O) tryptophan, amine site; (P) tryptophan, carboxylic site; (Q) histidine, amine site; (R) histidine, carboxylic site; (S) valine, amine site; (T) valine, carboxylic site. Gold atoms are represented in yellow, oxygen atoms in red, sulfur atoms in light yellow, hydrogen atoms in light pink, carbon atoms in brown, and nitrogen atoms in steel blue.

**Figure 7:**
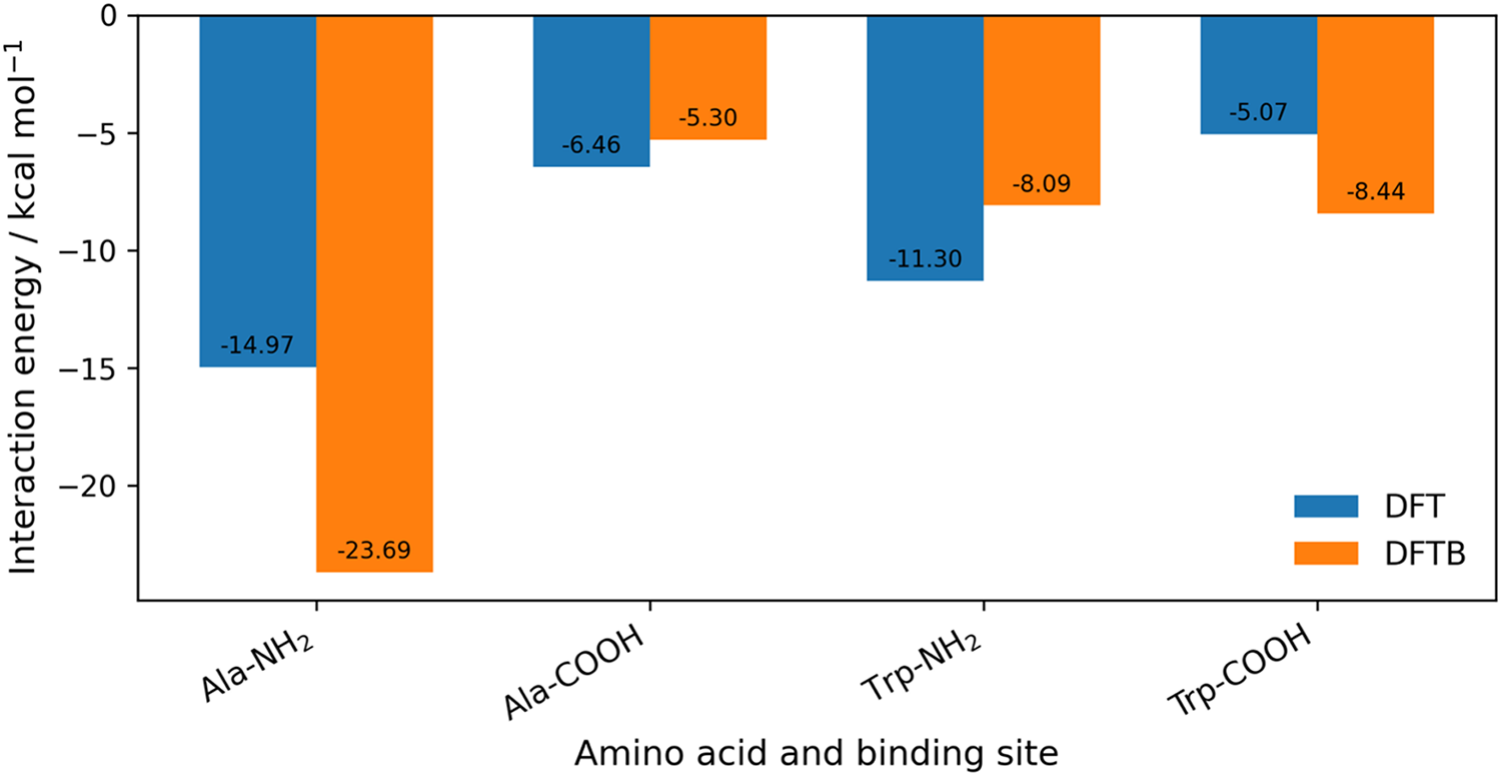
Comparison of DFTB and DFT interaction energies for Au_20_–amino acid complexes at the amine (NH_2_) and carboxyl (COOH) binding sites. Bars show the adsorption energies obtained with DFTB (orange) and DFT (blue) for alanine and tryptophan, the two amino acids for which DFT reference data are available for the Au_20_ cluster.

**Table 4:** Interaction Energies (*E*_int_, kcal/mol) and Au–X Bond Lengths (d, Å) for Au_20_–Amino Acid Complexes at Amine (X = N) and Carboxylic (X = O) Sites, as Obtained from DFTB Calculations

complex, interacting site	*E*_int_ (kcal/mol)	*d* (Au-X) (Å)
Au_20_-alanine, amine site	–23.69	2.88
Au_20_-alanine, carboxylic site	–5.30	3.02
Au_20_-asparagine, amine site	–12.07	2.85
Au_20_-asparagine, carboxylic site	–6.49	3.00
Au_20_-cysteine, amine site	–10.04	2.91
Au_20_-cysteine, carboxylic site	–4.59	3.00
Au_20_-glycine, amine site	–8.62	2.88
Au_20_-glycine, carboxylic site	–5.05	2.97
Au_20_-histidine, amine site	–13.93	2.91
Au_20_-histidine, carboxylic site	–4.63	3.03
Au_20_-phenylalanine, amine site	–18.98	3.00
Au_20_ -phenylalanine, carboxylic site	–6.20	2.97
Au_20_-proline, amine site	–11.18	2.92
Au_20_-proline, carboxylic site	–5.44	2.96
Au_20_-serine, amine site	–10.53	2.88
Au_20_-serine, carboxylic site	–6.13	2.97
Au_20_-tryptophan, amine site	–8.09	2.84
Au_20_-tryptophan, carboxylic site	–8.44	3.13
Au_20_-valine, amine site	–11.34	3.76
Au_20_-valine, carboxylic site	–5.65	2.95

The corresponding Au–X bond lengths predicted by DFTB follow the same trend. Amine-site Au–N bonds span 2.84 to 3.00 Å for most systems, with one exception for valine at 3.76 Å, while carboxyl-site Au–O distances range from 2.95 to 3.13 Å. In comparison, DFT reference values are systematically shorter by approximately 0.50–0.70 Å. For alanine, DFTB gives 2.88 Å at the amine site and 3.02 Å at the carboxyl site, while DFT^22^ reports 2.33 and 2.50 Å, corresponding to deviations of 0.55 and 0.52 Å. For tryptophan, the Au–N bond length is 2.84 Å from DFTB compared to 2.31 Å from DFT^22^, and the Au–O bond length is 3.13 Å compared to 2.45 Å, with deviations of 0.53 and 0.68 Å, respectively. Across all other amino acids, DFTB values cluster in the 2.90–3.00 Å range, suggesting systematic overestimation of absolute bond lengths while preserving relative differences between amine and carboxyl coordination.

These results indicate that Au_20_–amino acid interactions are qualitatively well described by DFTB, with amine binding consistently stronger and associated with shorter Au–N distances.

### Au_32_–Amino Acid Systems

For the Au_32_–amino acid complexes, Figure 8, DFTB predicts adsorption energies ranging from −4.64 to −23.70 kcal/mol at the amine site and from −6.17 to −14.30 kcal/mol at the carboxyl site. These values, along with data for all Au_32_-amino acid systems investigated, are reported in Table 5. Alanine shows the strongest stabilization at the amine site with −23.70 kcal/mol, while phenylalanine at −17.64 kcal/mol and histidine at −13.77 kcal/mol also bind relatively strongly. By contrast, glycine binds with −8.52 and −6.40 kcal/mol, cysteine with −8.34 and −7.24 kcal/mol, and tryptophan with −8.44 and −9.04 kcal/mol, indicating weaker interactions. Proline is a notable exception, exhibiting stronger binding at the carboxyl group with −14.30 kcal/mol compared to only −4.64 kcal/mol at the amine site. Serine shows comparable stabilization at both sites with −12.10 and −11.93 kcal/mol.

**Figure 8:**
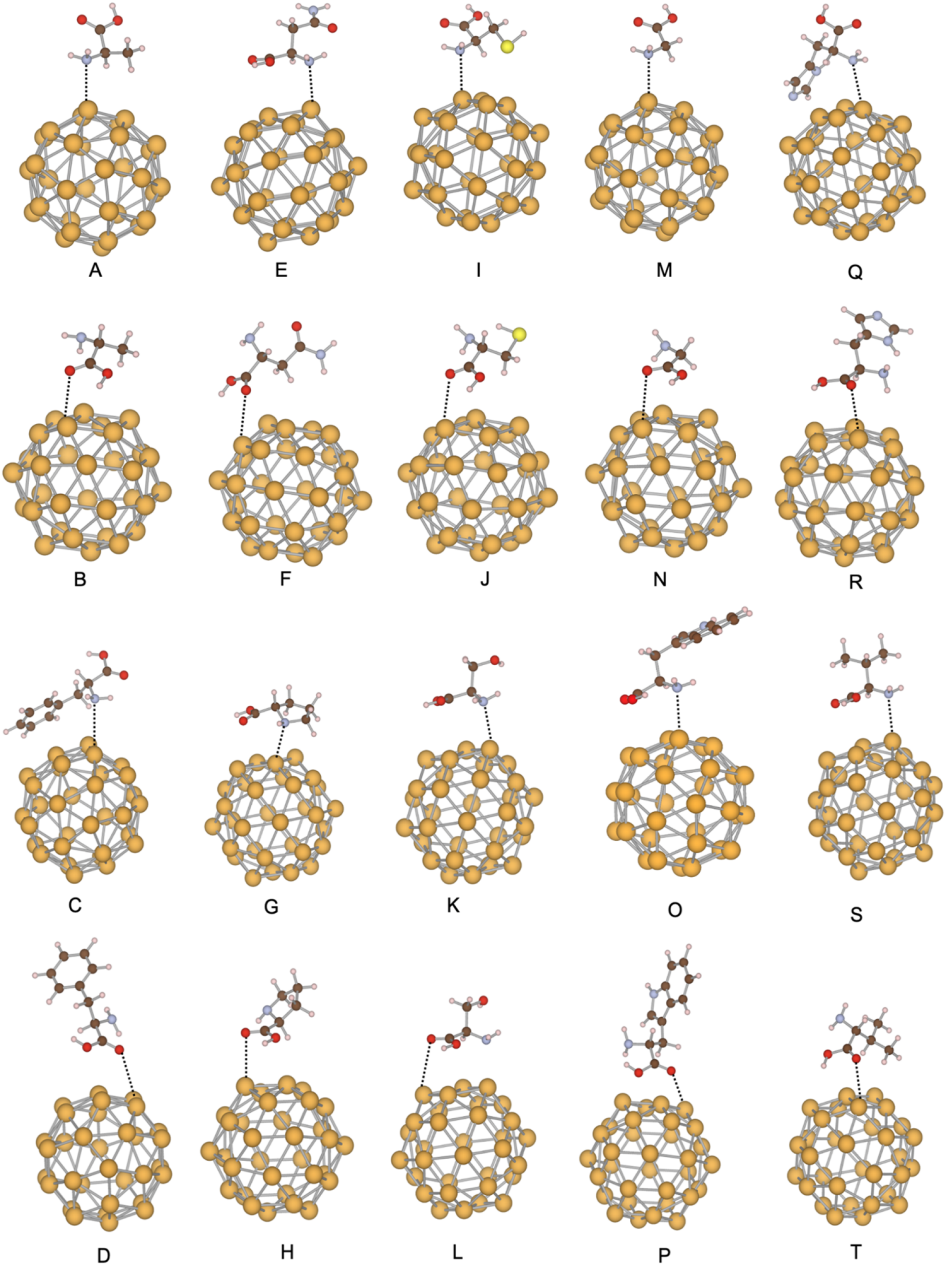
Local minima structures of the Au_32_ systems. The amino acids in each structure and interacting side are (A) alanine, amine site; (B) alanine, carboxylic site; (C) phenylalanine, amine site; (D) phenylalanine, carboxylic site; (E) asparagine, amine site; (F) asparagine, carboxylic site; (G) proline, amine site; (H) proline, carboxylic site; (I) cysteine, amine site; (J) cysteine, carboxylic site; (K) serine, amine site; (L) serine, carboxylic site; (M) glycine, amine site; (N) glycine, carboxylic site; (O) tryptophan, amine site; (P) tryptophan, carboxylic site; (Q) histidine, amine site; (R) histidine, carboxylic site; (S) valine, amine site; (T) valine, carboxylic site. Gold atoms are represented in yellow, oxygen atoms in red, sulfur atoms in light yellow, hydrogen atoms in light pink, carbon atoms in brown, and nitrogen atoms in steel blue.

**Table 5:** Interaction Energies (*E*_int_, kcal/mol) and Au–X Bond Lengths (d, Å) for Au_32_–Amino Acid Complexes at Amine (X = N) and Carboxylic (X = O) Sites, as Obtained from DFTB Calculations

complex, interacting site	DFTB *E*_int_ (kcal/mol)	DFTB *d* (Au-X) (Å)
Au_32_-alanine, amine site	–23.70	2.95
Au_32_-alanine, carboxylic site	–7.20	3.16
Au_32_-asparagine, amine site	–13.07	2.94
Au_32_-asparagine, carboxylic site	–9.11	3.35
Au_32_-cysteine, amine site	–8.34	3.02
Au_32_-cysteine, carboxylic site	–7.24	3.21
Au_32_-glycine, amine site	–8.52	2.97
Au_32_-glycine, carboxylic site	–6.40	3.36
Au_32_-histidine, amine site	–13.77	3.31
Au_32_-histidine, carboxylic site	–6.63	3.04
Au_32_-phenylalanine, amine site	–17.64	3.00
Au_32_ -phenylalanine, carboxylic site	–6.17	3.40
Au_32_-proline, amine site	–4.64	3.01
Au_32_-proline, carboxylic site	–14.30	3.28
Au_32_-serine, amine site	–12.10	3.64
Au_32_-serine, carboxylic site	–11.93	2.97
Au_32_-tryptophan, amine site	–8.44	3.05
Au_32_-tryptophan, carboxylic site	–9.04	3.27
Au_32_-valine, amine site	–11.82	2.98
Au_32_-valine, carboxylic site	–9.04	3.18

The Au–X bond lengths predicted by DFTB span 2.94–3.64 Å. Amine coordination generally yields Au–N distances shorter than those of carboxyl coordination, consistent with stronger adsorption, although the differences are less pronounced than those in smaller clusters. Alanine shows bond lengths of 2.95 Å at the amine site and 3.16 Å at the carboxyl site. Asparagine follows a similar trend with 2.94 and 3.35 Å. Deviations from this pattern include serine, which has a long Au–N distance of 3.64 Å at the amine site compared to 2.97 Å at the carboxyl site, and phenylalanine, where the carboxyl bond extends to 3.40 Å.

Overall, Au_32_ displays greater structural flexibility than smaller clusters, allowing alternative binding modes to become competitive. The reversal of the site preference for proline and the nearly equivalent stabilization of serine highlight the increasing complexity of the adsorption landscape at larger cluster sizes. Because no DFT data exist in the literature for these systems, it is not possible to evaluate the performance of DFTB. Nevertheless, the increased structural flexibility indicates that the quantitative accuracy of DFTB in this regime should be interpreted with care, as the method was parametrized primarily for smaller, more molecular-like clusters rather than fully metallic nanoparticles.

DFTB interaction energies of representative amino acids on gold clusters of increasing size are shown in Figure 9 for the selected set (Gly, Cys, Phe, His, Ser, and Val). These amino acids span the main classes of side-chain chemistry, including small reference systems (Gly), thiol-containing motifs (Cys), aromatic and heteroaromatic rings (Phe and His), polar O-donors (Ser), and branched hydrophobic groups (Val). The overall trends confirm that DFTB captures the qualitative evolution of adsorption strength with cluster size and functional-group type, while the comparison with available DFT reference data shows that quantitative deviations become more pronounced for larger clusters and for nitrogen-containing cyclic residues.

**Figure 9:**
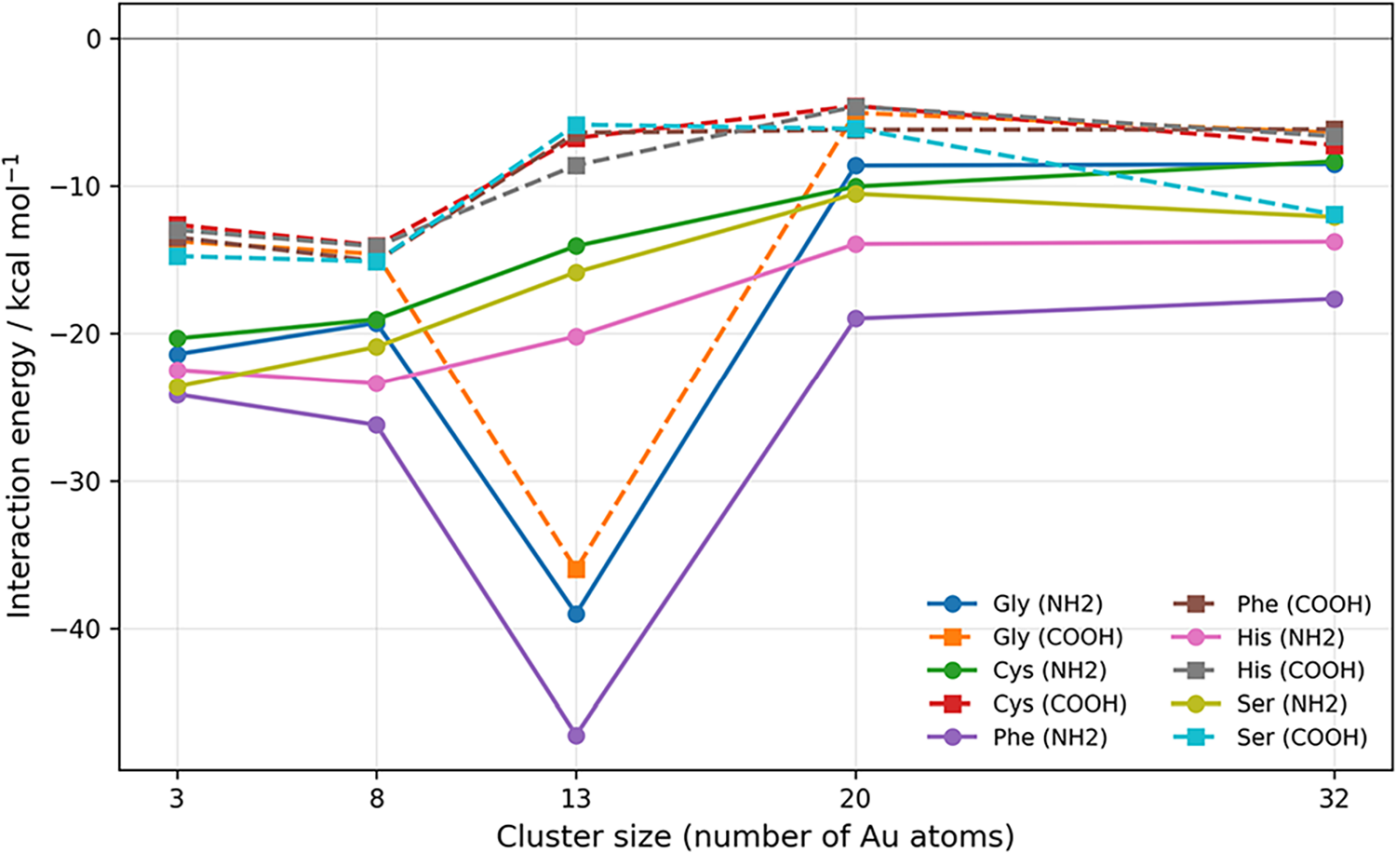
DFTB interaction energies of representative amino acids on gold clusters of increasing size. For each amino acid, two adsorption modes are shown: coordination at the amine site (solid line with circular markers) and coordination at the carboxyl site (dashed line with square markers).

## Conclusions

This study used DFTB with D3­(BJ) dispersion correction to describe the adsorption of amino acids on gold nanoclusters across a range of cluster sizes (Au_3_, Au_8_, Au_13_, Au_20_, and Au_32_). Interaction energies and Au–X bond lengths obtained with DFTB were compared with available DFT data, allowing an assessment of accuracy, transferability, and size-dependent trends. The DFTB parametrization employed here is based on scalar-relativistic DFT reference data for Au–X pairs, so the dominant relativistic effects associated with gold are implicitly included, while explicit spin–orbit coupling and higher-order relativistic corrections are not considered.

Overall, DFTB reproduces the qualitative binding preference of amino acids, with the amine site generally being more strongly bound than the carboxyl site. Quantitative agreement with DFT is good for several systems, with deviations typically within 2–3 kcal/mol for clusters such as Au_3_ and Au_8_. Systematic overestimation of bond lengths by ∼0.4–0.7 Å was observed, consistent across all cluster sizes. Larger deviations in interaction energies occur for certain side chains (notably nitrogen-containing cyclic systems), and Au_13_ shows enhanced variability due to its structural flexibility. These outliers reflect situations where polarization, partial charge transfer, and ligand-induced reconstructions become more important, pushing the DFTB approximation beyond the regime for which its Au–X parameters were primarily optimized.

As the cluster size increases from Au_3_ and Au_8_ to Au_13_, Au_20_, and Au_32_, DFTB continues to capture qualitative trends in site preference and side-chain dependence, but quantitative deviations from DFT increase because pairwise parametrization and simplified treatment of polarization and many-body dispersion do not fully reproduce the more delocalized and many-electron nature of larger clusters. Consequently, the present benchmarks should be regarded as a size-dependent validation up to Au_32_, rather than as a universal calibration for arbitrarily large Au nanoparticles. Dispersion interactions play a significant modulatory role in these systems. While the primary contribution to binding arises from Au–X (X = C, O) coordination, van der Waals forces provide additional stabilization that is especially important for bulky, polarizable side chains such as phenylalanine and tryptophan. The D3­(BJ) correction used here offers an efficient, pairwise description of this contribution, but it does not account for many-body dispersion effects that may become more relevant as cluster size and aromatic surface area increase.

These results demonstrate that DFTB provides a computationally efficient and reasonably accurate framework for exploring Au–biomolecule interactions. At the same time, careful benchmarking against higher-level methods remains essential for quantitative predictions, particularly for clusters prone to significant structural rearrangement or for amino acids with more complex binding motifs. In particular, nitrogen-containing cyclic residues and strongly deformable cluster sizes such as Au_13_ represent challenging test cases in which DFTB should be applied with additional caution and, where possible, validated directly against DFT or higher-level electronic structure methods.

For the systems in which DFTB and DFT show good agreement, we plan to extend this work by investigating how amino acid adsorption affects the band structure, the role of explicit solvent effects, and changes in electrostatic potentials, charge distribution, and orbital character. In future work, we also intend to examine changes in frontier orbital energies and highest occupied molecular orbital–lowest-unoccupied molecular orbital (HOMO–LUMO) gaps upon adsorption, in order to connect the structural trends reported here with reactivity descriptors and potential implications for electron transfer and catalytic activity. Molecular dynamics simulations will also be employed to capture conformational flexibility and thermodynamic stability under more realistic conditions. In addition, global structure search strategies will be incorporated to better explore the configurational space of Au–amino acid complexes, ensuring that the most stable adsorption motifs are identified before detailed property analysis.
